# *In vitro* Antimycobacterial, Apoptosis-Inducing Potential, and Immunomodulatory Activity of Some Rubiaceae Species

**DOI:** 10.3389/fphar.2019.00185

**Published:** 2019-03-05

**Authors:** Abimbola O. Aro, Jean Paul Dzoyem, Amelia Goddard, Pascaline Fonteh, Prudence N. Kayoka-Kabongo, Lyndy J. McGaw

**Affiliations:** ^1^Phytomedicine Programme, Department of Paraclinical Sciences, Faculty of Veterinary Science, University of Pretoria, Pretoria, South Africa; ^2^Department of Agriculture and Animal Health, College of Agriculture and Environmental Sciences, University of South Africa, Florida, South Africa; ^3^Department of Biochemistry, Faculty of Science, University of Dschang, Dschang, Cameroun; ^4^Department of Companion Animal Clinical Studies, Faculty of Veterinary Science, University of Pretoria, Pretoria, South Africa; ^5^Department of Surgery, Faculty of Health Sciences, University of the Witwatersrand, Johannesburg, South Africa

**Keywords:** antimycobacterial, tuberculosis, *M. tuberculosis* complex, Rubiaceae, immunomodulatory, apoptosis, lipoxygenase, cytokines

## Abstract

Tuberculosis (TB), a disease caused by microorganisms of the *Mycobacterium tuberculosis* complex, infects almost one-third of the world’s population. The TB epidemic has been further exacerbated by the emergence of multi, extensively, and totally-drug-resistant (MDR, XDR, and TDRTB) strains. An effective immune response plays a crucial role in determining the establishment of TB infection. Therefore, the modulation of the immune system has been considered as a vital approach for the treatment or control of various immune-related diseases such as TB. In this study, the antimycobacterial, immunomodulatory, and apoptosis-inducing effects of six Rubiaceae species were evaluated. A twofold serial dilution method was used to determine the minimum inhibitory concentration values of the plant extracts. The effect of the extracts on the activity of 15-lipoxygenase was investigated. The levels of six different cytokines, IL-2, IL-4, IL-5, IL-10, IFN-γ, and TNF-α, were measured in LPS-activated U937 cell line while the apoptosis-inducing effect of the extracts was evaluated using an annexin V/PI assay using a flow cytometer. The results obtained revealed that all the six extracts tested had antimycobacterial activity against *M. tuberculosis* H37Rv, *M. tuberculosis* ATCC 25177, and *Mycobacterium bovis* ATCC 27299 strains, with MIC values ranging from 39 to 312 μg/mL. The extracts of *Cremaspora triflora* and *Cephalanthus natalensis* were the most active against *M. tuberculosis* (MIC = 39 μg/mL), followed by *Pavetta lanceolata* and *Psychotria zombamontana* against *M. bovis* (MIC = 78 μg/mL). The extracts of *P. zombamontana* and *Psychotria capensis* had remarkable IC_50_ values of 4.32 and 5.8 μg/mL, respectively, better than that of quercetin. The selected extracts promoted Th1/Th2 balances in an *in vitro* model at the tested concentration which may suggest the therapeutic value of the plant in diseases where inflammation is a significant factor such as TB. The addition of the crude extracts of *C. triflora*, *P. capensis*, and *P. zombamontana* at the tested concentrations to the cell culture medium induced apoptosis in a time- and dose-dependent manner. This interesting preliminary result generated from this study encourages further investigations of these extracts owing to the LOX-inhibitory effect, immunomodulatory, and apoptotic-inducing properties in addition to their antimycobacterial properties.

## Introduction

Tuberculosis, a disease caused by organisms belonging to the *Mycobacterium tuberculosis* complex, resulted in the death of 1.5 million people and approximately 9.6 million new cases in 2014 (Anderson et al., 2015). TB, an old disease that has plagued humankind for centuries, is known to be a highly infectious disease and still remains the leading killer among infectious diseases in the world, with approximately 2 billion people being latently infected with this obligate parasite (Nguta et al., 2015; World Health Organization [WHO], 2017). The TB epidemic has been further exacerbated by the emergence of MDR-TB, XDR-TB, and TDR-TB strains (Riccardi and Pasca, 2014; Blanco et al., 2015; World Health Organization [WHO], 2017).

An effective immune response to *M. tuberculosis* plays a crucial role in determining the establishment of disease (Kleinnijenhuis et al., 2011). This facultative pathogen has devised various mechanisms such as immune-evasion strategies to subvert the host immune response (Saini et al., 2016). Also, the intricate interaction of *M. tuberculosis* with the immune system leads to the release of a vast array of cytokines by diverse cell types in response to infection (Etna et al., 2014). The mechanisms used by *M. tuberculosis* to modify macrophage functions have not been fully elucidated. The interaction between mycobacteria and cells of both the innate and adaptive immune system results in the secretion of chemokines and cytokines, the most important being tumor necrosis factor-α (TNFα), cytokines of the interleukin-1 family (IL-1β, IL-18), IL-12, and IFN-γ (Kleinnijenhuis et al., 2011). The success of pathogenic mycobacteria is largely attributed to their capacity to avoid destruction within host immune cells.

Lipid mediators such as eicosanoids and PGEs implicated in inflammation have emerged as potential therapeutic agents. Leukotrienes and lipoxins (LXAs) are generated from AA by 5- and 15-LOX while PGEs are generated from AA by COX I and II. However, after infection with mycobacteria, the balance of PGEs and LXAs determines the apoptotic and necrotic effect with PGEs preventing necrosis and inducing apoptosis while LXAs promote necrosis (Behar et al., 2010; Divangahi et al., 2013). The *M. tuberculosis* complex species delay the initiation of adaptive immunity by inhibiting apoptosis and initiating the necrotic effect of host macrophages (Divangahi et al., 2010). The apoptotic response represents an innate defense mechanism against intracellular mycobacteria (Fratazzi et al., 1997). Ultimately, host macrophage apoptosis but not necrosis is linked to killing of intracellular mycobacteria (Molloy et al., 1994). This suggests that programmed cell death of the host macrophage not only eliminates a preferred growth niche for *M. tuberculosis* but also activates a unique microbicidal mechanism. Therefore, the modulation of the immune system has been considered a vital approach for the treatment or control of various immune-related diseases such as TB (Cho, 2008; Ouchi et al., 2011). In addition to the drug development process of novel anti-TB chemotherapy, it is imperative to harness advances in systems-level analysis of host–pathogen interactions; a new approach referred to as adjunct therapy (Worthington and Melander, 2013). In TB chemotherapy, the microbicidal activity of anti-TB drugs alone is not sufficient but should include modulation of the host immune response pathways in order to combat the causative pathogen of the disease, thereby synergistically enhancing the activity of the drugs (Rayasam and Balganesh, 2015).

An increasing number of studies have shown that traditional phytomedicine confer a variety of immunomodulatory activities (Hou et al., 2010; Du et al., 2013). Therefore, plant products that possess immunomodulatory activities have potential as therapeutic agents for various inflammatory diseases (Devasagayam and Sainis, 2002; Nowakowska, 2007). There are 611 genera in the Rubiaceae family but only 48 genera have been studied and reported to have excellent antibacterial activity against various pathogenic strains (Choudhury et al., 2012). Many molecules isolated from some genera belonging to the Rubiaceae family are pertinent in drug discovery due to the fact that they serve as templates in the drug development process (Ngwenya, 2008). There are 61 genera and 228 species native or naturalized in southern Africa (Ngwenya, 2008). In this study, six species from the Rubiaceae family, namely, *Cephalanthus natalensis*, *Cremaspora triflora*, *Oxyanthus speciosus*, *Pavetta lanceolata*, *Psychotria capensis*, and *Psychotria zombamontana*, with good antimycobacterial activity (Aro et al., 2015) were chosen for further study to determine their LOX-inhibitory effect, immune modulatory, and apoptotic inducing properties, in an effort to gain insight into their potential mechanisms of action.

## Materials and Methods

### Preparation of Plant Extracts and Thin Layer Chromatography Analysis

The leaves of the six plant species were collected in November 2009 from the University of Pretoria (PRU) Botanical Gardens, Pretoria, South Africa, and Lowveld National Botanical Gardens (Nelspruit). The plant materials were labeled and identified by Magda Nel from the Department of Plant Sciences, PRU. Voucher specimens were kept in the HGWJ Schweickerdt Herbarium of the PRU. The leaves were air dried at room temperature, ground to a fine powder in a Macsalab mill (model 2000 LAB Eriez), and stored in closed glass containers in the dark until needed. The powdered plant material was extracted with acetone in a ratio of 1:10 of plant material to acetone (technical grade, Merck) in a polyester centrifuge tube which was vigorously shaken on an orbital shaker for 30 min (Eloff, 1998). It was centrifuged at 4000 × *g* for 10 min and the supernatant was filtered through Whatman No. 1 filter paper into a pre-weighed glass vial. The extraction was repeated twice on the same plant material and the solvent was removed under a stream of air in a fume hood at room temperature to produce dried extracts. Extracts were reconstituted to a concentration of 10 mg/mL in acetone. The acetone crude extracts were qualitatively screened to obtain thin layer chromatography (TLC) fingerprints of each investigated extract; 10 μL from 100 μg of extract stock was preloaded on aluminum-backed TLC plates (Merck, Silica gel F254) in lines of about 1 cm wide. Three different mobile solvent systems which include ethyl acetate/methanol/water (EMW) 10:1.35:1, chloroform/ethyl acetate/formic acid (CEF) 10:8:2, and benzene/ethanol/ammonia (BEA) 18:2:0.2 were used to develop the chromatogram on the TLC plates (Kotze et al., 2002). The developed chromatograms were sprayed with a mixture of vanillin (0.1 g) dissolved in methanol (28 mL) and sulfuric acid (1 mL), heated to 110°C for optimal color development and were examined under ultraviolet light at wavelengths of 254 and 365 nm.

### Antimycobacterial Assay

Antimycobacterial activity was tested against three mycobacterial species, namely, *M. tuberculosis* H37Rv, *M. tuberculosis* (ATCC 25177), and *Mycobacterium bovis* (ATCC 27290). The mycobacterial species were cultured on Löwenstein–Jensen agar slants, supplemented with glycerol, or pyruvate in the case of the *M. bovis* culture. To avoid formation of clumps, sterile plastic loops were used to scrape cells off the slants prior to each assay. These suspensions were diluted with sterile water to adjust the turbidity to a No 1 McFarland standard (approximately 4 × 10^7^ cfu/mL), and then diluted with freshly prepared Middlebrook 7H9 medium (Difco, Becton Dickinson, United States) supplemented with 10% oleic acid-albumin-dextrose-catalase (OADC, Becton Dickinson, United States) and 0.5% glycerol (Merck Millipore, Germany) to obtain a final inoculum density of approximately 5 × 10^5^ cfu/mL.

A twofold serial dilution method was used to determine the minimum inhibitory concentration (MIC) values of the plant extracts (Eloff, 1998; McGaw et al., 2008). Crude extracts were dissolved in 10% DMSO to prepare stocks of 10 mg/mL. The assay was carried out in 96-well plates. Briefly, 100 μL of the stock solutions of the crude extracts were serially diluted in 100 μL of OADC-supplemented Middlebrook 7H9 broth in 96-well microtiter plates before mycobacterial culture (100 μL) was added to each well. The anti-TB drugs rifampicin and streptomycin represented the positive controls, and solvent controls were included. Doses were tested at least in triplicate and the entire experiments were repeated three times. Plates were incubated at 37°C for 10–14 days. MIC values were observed using a tetrazolium violet (INT) indicator. This experiment was carried out in a Biosecurity Level 2+ containment facility with appropriate personal protective equipment.

### Soybean Lipoxygenase Inhibition Assay

The assay was performed according to a previously described procedure (Pinto et al., 2007) with slight modifications described by Dzoyem and Eloff (2015). The LOX inhibitory activity was evaluated by calculating the percentage of the inhibition of hydroperoxide production from the changes in absorbance values at 560 nm after 30 min at 25°C.

$$\%\text{ inhibition}=[({\text{A}}_{\text{control}}-{\text{A}}_{\text{blank}})-({\text{A}}_{\text{sample}}-{\text{A}}_{\text{blank}})/({\text{A}}_{\text{control}}-{\text{A}}_{\text{blank}})]\times 100$$

where A_control_ is the absorbance of the control well, A_blank_ is the absorbance of the blank well, and A_sample_ is the absorbance of the sample well.

### Cytotoxicity Assay

The cytotoxicity of the crude plant extracts was tested against human monocytic cell line THP-1 (purchased from ATCC, TIB-202) and human promonocytic cell line U937 (ATCC 1593.2) using the 3-(4,5-dimethylthiazol)-2,5-diphenyl tetrazolium bromide (MTT) assay (Mosmann, 1983) with slight modifications (McGaw et al., 2007).

### Immunomodulatory Assay

The concentration used for determining immunomodulatory activity of the plant extracts was at their IC_50_ value (the 50% inhibitory concentration values which were calculated as the concentration of test compound resulting in a 50% reduction of absorbance compared to untreated cells). The anti-tubercular drug, rifampicin, was also included at the LC_50_ value. Each sample concentration was freshly prepared on the day of experiment in RPMI medium with acetone as acetone is not toxic to the cell line at the percentage tested, while rifampicin was prepared in DMSO (0.2%).

The human promonocytic cell line U937 (ATCC 1593.2) available from Highveld Biological (Pty) [(pH 7.2), was cultured in medium containing 10% heat inactivated fetal calf serum (FCS), 2 mM l-glutamine, and a 0.1% antimicrobial solution consisting of penicillin, streptomycin, and fungizone]. Reagents were procured from Highveld Biological (Pty) (Ltd.) (Sandringham, South Africa). U937 cell suspension (2 × 10^5^ cells/mL) was seeded into 96-well tissue culture microtiter plates supplemented with 0.10 μg/mL PMA (Sigma) for 48 h at 37°C in an atmosphere of 5% CO_2_ to induce differentiation into macrophage-like cells (Passmore et al., 2001). After the incubation period, the differentiated U937 cells were washed with phosphate-buffered saline (PBS) (Lonza, South Africa). The PMA-containing medium was replaced with PMA-free RPMI-1640 medium after 48 h incubation. Cytotoxicity of the extracts against U937 cells was determined as described by Mosmann (1983) and modified by McGaw et al. (2007).

The differentiated cell suspension was stimulated with LPS (5 μg/mL) and treated with increasing concentrations of crude extracts and rifampicin. Puromycin (5.0–0.1 μM) was used as a positive control because it can prevent growth of bacteria, protozoa, algae, and mammalian cells and acts quickly, killing 99% of cells within 2 days at 1–10 μM (Sigma). After 48 h incubation in a CO_2_ incubator, the supernatant was collected and stored at −80°C. To determine the viability of the cells, the cells were then washed with PBS, fresh medium (200 μL) was added, and 30 μL of MTT (5 mg/mL in PBS) was added to each well and the plates were incubated at 37°C for 4 h. The medium was removed by aspiration and 100% DMSO (50 μL) added to dissolve the resulting formazan crystals. The absorbance was measured on a BioTek Synergy microtiter plate reader at 570 nm. The percentage of cell growth inhibition was calculated by comparison with untreated cells.

### Cytokine Detection via Cytometric Bead Array (CBA) Analysis

Cell culture supernatants were thawed once and examined for IL-2, IL-4, IL-5, IL-10, IFN-γ, and TNF-α concentrations by multiplex cytokine array analysis performed using the cytometric bead array (CBA) method using the Human Th1/Th2 Kit (BD-Biosciences). The six capture beads were mixed on a Vortex mixer and 50 μL was dispensed into each of the assay tubes. Fifty microliters of the relevant phycoerythrin (PE) detection reagent was then added to the assay tubes. For each of the test samples, 50 μL of cell supernatant was added to the test assay tubes and 50 μL of the cytokine standard dilutions were added to the control assay tubes and were incubated at room temperature for 3 h away from sunlight. During the incubation, the cytometer setup procedure was performed. After the incubation time, 1 mL of wash buffer was added to each assay tube and centrifuged at 200 × *g* for 5 min. To avoid disturbing the bead pellet, the supernatant from each assay tube was carefully aspirated and discarded. Thereafter, 300 μL wash buffer was added to each assay tube to resuspend the bead pellet and mixed thoroughly on a vortex for 5 s before plating the samples in a labeled 96-well microtiter plate. Acquisition was performed with flow cytometry utilizing the BD Accuri C6 and the data were analyzed with the FCAP Array software. The sensitivity for each cytokine using the BD CBA Human Th1/Th2 Cytokine Kit is as follows: IL-2: 3.30 pg/mL; IL-4: 1.87 pg/mL; IL-5: 0.76 pg/mL; IL-10: 0.66 pg/mL; TNF-α: 1.61 pg/mL; IFN-γ: 7.79 pg/mL. These theoretical limits of detection are defined as the corresponding concentration at two standard deviations above the median fluorescence of 20 replicates of the negative control (0 pg/mL).

### Apoptosis Assay Using Flow Cytometry

The human monocytic cell line THP-1 (purchased from ATCC, TIB-202) was cultured in RPMI-1640 (containing L-glutamine and nabicarbonate; Sigma, United States) supplemented with 10% FCS (Highveld Biological, South Africa) and 1% antibiotics comprising streptomycin and penicillin (Highveld Biological, South Africa). Cells were seeded into 24-well flat bottomed plates (Nest) at 2 × 10^5^ cells/well. After 24 h of incubation, cells were exposed to different concentrations (20, 50, and 100 μg/mL) of plant extracts for 24 and 48 h. The crude extracts of *C. natalensis* were not included due to insufficient plant material.

Wells containing cells only were included as control while cells exposed to DMSO only were included as vehicle control. Staurosporine (0.2 μM) was included as positive control. Cells were harvested at different time points and then washed twice with warm PBS. Apoptosis was measured using the Annexin V-fluorescein isothiocyanate (FITC)/propidium iodide (PI) apoptosis detection kit (BD Bioscience) as per manufacturer’s instruction. Following 24 and 48 h of incubation (5% CO_2_, 37°C) of the cells exposed to plant extracts, the percentage of apoptotic cells was determined by the annexin V-FITC/PI assay.

Cells were harvested and transferred into plastic flow tubes (BD Biosciences, South Africa), washed with 1 mL cold PBS, centrifuged at 250 × *g* for 5 min and resuspended in 100 μL of binding buffer. The cells were stained with 2 μL of annexin V-FITC conjugate and PI, vortexed and incubated on ice in the dark for 15 min followed by addition of 250 μL of cold binding buffer to stop the reaction. The annexin positive, the PI positive, as well as unstained cell controls were used for compensation and quadrant specification. The controls and samples suspended in 500 μL of binding buffer were analyzed by flow cytometry using BD LSRFortessa software (BD Biosciences, South Africa) within 30 min after staining, with 10,000 events collected for each sample. The data acquisition was performed using FACSDiva software while analysis of the results was processed with FlowJo 10.1.

### Statistical Analysis

All experiments were conducted in triplicate and values expressed as mean ± standard deviation. Statistical analysis was performed using two-way ANOVA. Significant differences between treated and control were determined with Dunnett’s tests using GraphPad Prism 6. (GraphPad Software, Inc., La Jolla, CA, United States).

## Results

### Antimycobacterial, Lipoxygenase Inhibition, and Cytotoxicity Activities

Based on the twofold serial microdilution assay, all the tested crude extracts had good to moderate MIC values ranging from 39 to 312 μg/mL against the tested pathogenic strains. The acetone extract of *C. triflora* had the best activity against *M. tuberculosis* with an MIC value of 39 μg/mL (Table 1). The crude extracts of *P. lanceolata* and *P. zombamontana* had a good MIC value of 78 μg/mL against *M. bovis* while *C. natalensis* and *O. speciosus* also had MIC value of 78 μg/mL against *M. tuberculosis* H37Rv.

**Table 1:** Antimycobacterial (MIC μg /mL), cytotoxicity (IC_50_ μg /mL), and anti-lipoxygenase (IC_50_ μg /mL) activities of the tested extracts.

Samples	MIC (μg /mL)			IC_50_		
	*H37Rv*	*M. tb*	*M. bovis*	U937	THP-1	LOX
*Cephalanthus natalensis* Oliv.	**78**	156	156	100 ± 0.01	100 ± 0.01	32.45 ± 1.05
*Cremaspora triflora* Thonn.	156	**39**	156	100 ± 0.10	378 ± 0.21	29.86 ± 2.39
*Oxyanthus speciosus* DC.	**78**	**78**	312	190 ± 0.00	600 ± 0.01	11.20 ± 7.30
*Pavetta lanceolata* Eckl.	156	156	**78**	125 ± 0.36	188 ± 0.03	10.85 ± 0.83
*Psychotria capensis* Vatke	156	312	156	**25 ± 0.00**	**761 ± 0.10**	5.8 ± 1.94
*Psychotria zombamontana* Kuntze	156	312	**78**	400 ± 0.00	579 ± 0.57	4.32 ± 1.10
Rifampicin	0.05	0.07	0.19	>200 ± 0.02	nd	nd
Streptomycin	0.1	0.5	0.1	nd	nd	nd
Puromycin	nd	nd	nd	4 ± 0.02	3.6 ± 0.01	nd
Quercetin	nd	nd	nd	nd	nd	25.53 ± 1.18

*nd: not determined; LOX: 15-lipoxygenase. Values written in bold are those with good antimycobacterial activities and low cytotoxicity.*

The 15-LOX inhibiting activity was measured using the 96-well microplate-based ferric oxidation of xylenol (FOX) orange assay. The selected plant extracts had IC_50_ values between 4.32 and 32.45 μg/mL indicating moderate to high LOX inhibitory activity when compared to the positive control quercetin with an IC_50_ of 25.45 μg/mL. The crude extracts of *P. zombamontana* and *P. capensis* had the best LOX inhibitory effect with IC_50_ of 4.32 and 5.8 μg/mL, respectively, better than that of quercetin (Table 1).

Based on the phytochemical analysis conducted, compounds of varying polarities were visualized on the TLC plate when sprayed with vanillin. More compounds were visible in BEA; a non-polar solvent followed by CEF; an intermediate polar solvent. The extracts poorly separated in EMW polar solvent (Supplementary Data).

Cytotoxicity was determined after supernatant collection using the MTT colorimetric method. Extracts showing sensitivity to cell lines with IC_50_ values > 100 μg/mL are considered not cytotoxic (Kuete, 2010). Five out of the six tested plant extracts had relatively low cytotoxicity (LC_50_ values ranging from 100 to 400 μg/mL) against the tested cell line except for the crude extract of *P. capensis.* This extract was cytotoxic against the U937 cell line with an IC_50_ value of 25 μg/mL (Table 1), but surprisingly it was non-cytotoxic to the THP-1 cells. Alkaloids are cytotoxic to various cell lines and have been isolated from the *Psychotria* genus (Martins et al., 2013). The cytotoxicity of the extract of *P. capensis* against U937 cells could be due to the presence of alkaloids. Interestingly, none of the tested crude extracts was cytotoxic to the THP-1 cell line with an IC_50_ values ranging from 188 to 761 μg/mL.

### Immunomodulatory Activity

**FIGURE 1:**
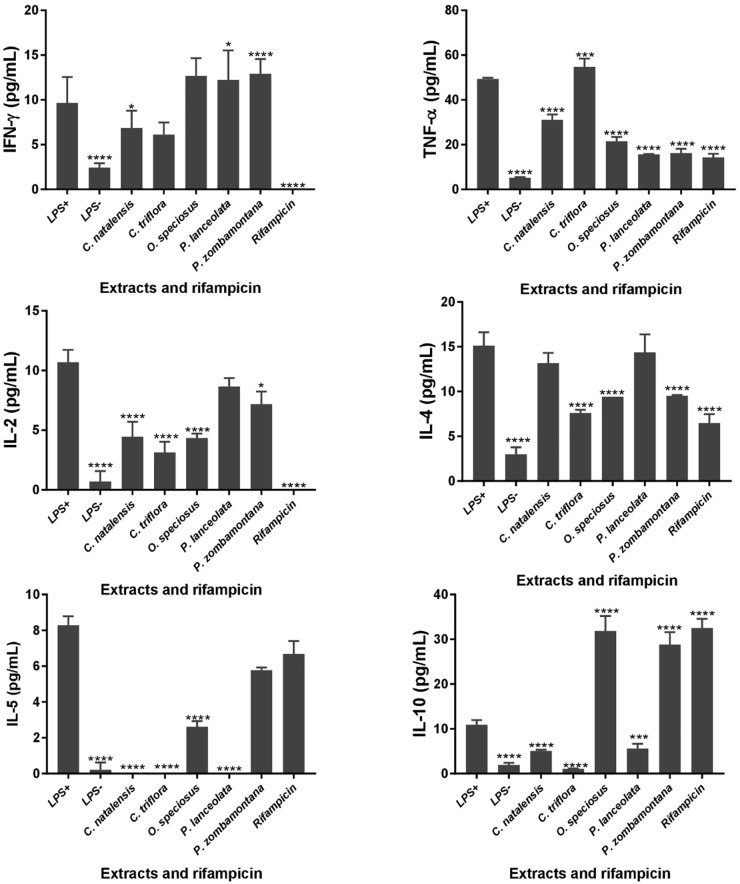
Controls: cells not stimulated with LPS (LPS -); cells stimulated with LPS, no treatment (LPS +). Effects of cytokine production in LPS-stimulated U937 cells treated for 48 h with varying concentration of five Rubiaceae crude extracts and rifampicin. Data are mean ± SD value. Statistical analysis was performed using Dunnett’s multiple comparisons test using two-way ANOVA. ^∗^*P* < 0.05, ^∗∗∗^*P* < 0.001, ^∗∗∗∗^*P* < 0.0001 versus LPS-stimulated cells only.

The levels of six different cytokines, IL-2, IL-4, IL-5, IL-10, IFN-γ, and TNF-α were measured using the CBA assay, a particle-based immunoassay combined with flow cytometry. LPS-stimulated U937 cells were treated with different concentrations of acetone crude extracts and rifampicin and the supernatants were collected after 48 h of exposure (Figure 1). *P. capensis* extracts were not included due to the cytotoxic effect observed on the cell line. Most of the Th1/Th2 cytokine secretion levels in the absence or presence of LPS was higher than the detection limits from the manufacturer (IL-2: 3.30 pg/mL; IL-4: 1.87 pg/mL; IL-5: 0.76 pg/mL; IL-10: 0.66 pg/mL; TNF-α: 1.61 pg/mL; IFN-γ: 7.79 pg/mL). The crude extracts of *P. zombamontana* significantly increased the expression of IFN-γ (12.91 pg/mL; *P* < 0.0001) while the expression observed for *O. speciosus* and *P. lanceolata* was not significant (*P* > 0.05). However, rifampicin significantly (*P* < 0.0001) inhibited the expression of this Th1 cytokine; IFN-γ compared to LPS-activated cells (Figure 1).

All the extracts significantly decreased the production of TNF-α (*P* < 0.0001) with the exception of *C. triflora* (54.69 pg/mL; *P* < 0.001) having a stimulatory effect relative to LPS-stimulated control. Also, acetone extracts of *C. natalensis*, *C. triflora*, *O. speciosus*, and rifampicin significantly suppressed the expression of pro inflammatory cytokine IL-2 (*P* < 0.0001). The inhibitory effect of the extracts of *C. triflora* and *O. speciosus* on Th2 cytokines; IL-4 and IL-5 was of statistical significance (*P* < 0.0001) when compared to LPS-stimulated cells except for *P. zombamontana* and rifampicin showing non-significant inhibitory effect of IL-5 (*P* > 0.05). Surprisingly, the expression of IL-10 was of significance for the extracts of *O. speciosus* (31.84 pg/mL), *P. zombamontana* (28.80 pg/mL), and rifampicin (32.50 pg/mL) all having *P*-values < 0.0001 while a significant decrease was observed for the extracts of *C. natalensis* (5.08 pg/mL), *C. triflora* (1.07 pg/mL), and *P. lanceolata* (5.59 pg/mL).

### Apoptosis Induction

**FIGURE 2:**
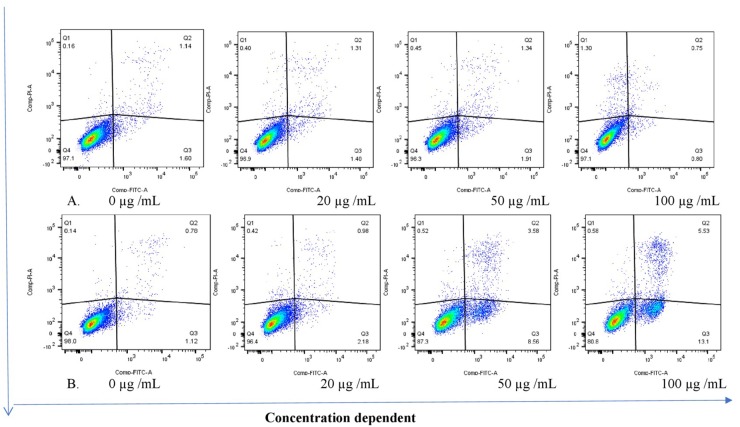
Stained cells (untreated control) and crude extract of *P. capensis* after **(A)** 24 and **(B)** 48 h of treatment induced apoptosis of THP-1 cells. Q1: necrotic cells (annexin V-/PI+); Q2: late apoptosis (annexin V+/PI+); Q3: apoptotic cells; (annexin V+/PI-) Q4: live cell (annexin V-/PI-).

**FIGURE 3:**
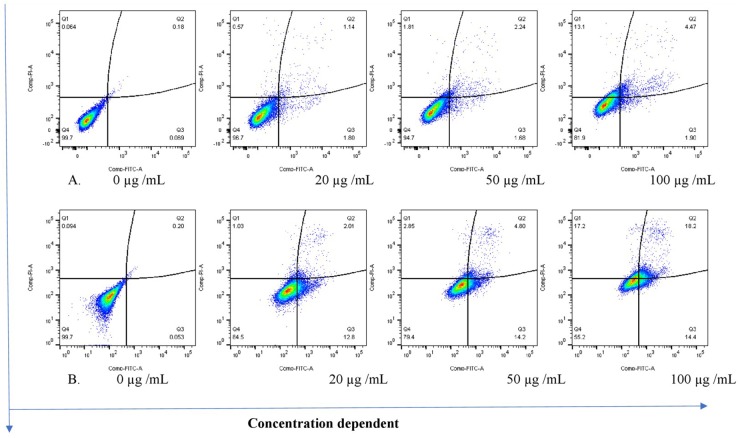
Stained cells and crude extract of *C. triflora* induces apoptosis of THP-1 cells post **(A)** 24h and **(B)** 48 h of treatment. Q1: necrotic cells (annexin V-/PI+); Q2: late apoptosis (annexin V+/PI+); Q3: apoptotic cells; (annexin V+/PI-) Q4: live cell (annexin V-/PI-).

**FIGURE 4:**
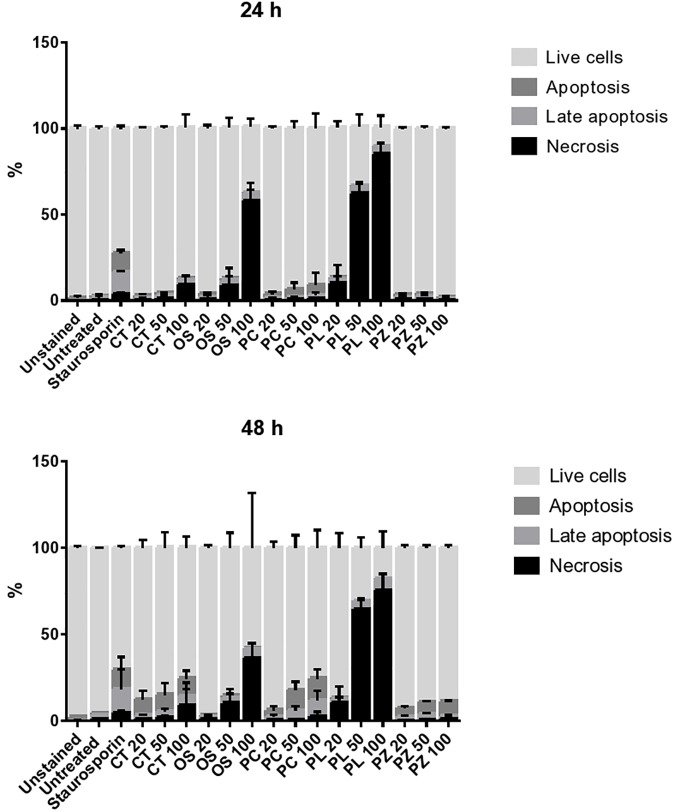
Evaluating the mode of cell death of THP-1 cell macrophages treated for 24 and 48 h with different concentration of plant extracts. Staurosporine (0.2 μM) was used as positive control. The bar represents the mean ± SD of a representative experiment among three independent experiments. CT = *Cremaspora triflora*, OS = *Oxyanthus speciosus*, PC = *Psychotria capensis*, PL = *Pavetta lanceolata*, PZ = *Psychotria zombamontana*.

The THP-1 cells were exposed to varying concentrations of five of the plant extracts for either 24 or 48 h and apoptosis was measured using the annexin V-FITC/PI assay. However, the extracts of *C. natalensis* could not be assessed for its apoptosis inducing effects due to insufficient plant material. The treatment of THP-1 cells with the acetone extracts of five different Rubiaceae species led to an increase in the percentage of annexin V positive apoptotic cells at 24 and 48 h in comparison to the percentage of cells without treatment (control or 0 μg/mL) in a dose- and time-dependent manner. It was observed that the addition of the crude extracts of *C. triflora* and *P. capensis* at 20, 50, and 100 μg/mL to the cell culture medium induced apoptosis in a time- and dose-dependent manner (Figure 2, 3). A necrotic effect (65–85%) higher than that of the positive control (3%) was observed for the extracts of *O. speciosus* and *P. lanceolata* at 50 and 100 μg/mL while at a non-cytotoxic concentration (20 μg/mL), a lower necrotic effect (<1%) was observed as depicted in Figure 4 which is a bar graph representing the cumulative results of five of the extracts.

## Discussion

Tuberculosis is a re-emerging infectious disease causing considerable mortality as well as morbidity. Pathogenic *Mycobacterium* spp. have developed resistance to many antibiotics and this situation has called for an urgent need to develop new anti-TB drugs from plants. Plants from the Rubiaceae family have been reported to exhibit antimalarial, antimicrobial, antihypertension, antidiabetic, antioxidant, and anti-inflammatory activities (Sirigiri, 2015). We have previously reported pharmacological activities of some South African Rubiaceae species including antioxidant, anti-inflammatory, synergistic effect, and antimycobacterial activities against pathogenic tuberculous isolates and fast growing non-tuberculous *Mycobacterium* species (Aro et al., 2015, 2016). Following our earlier findings of antimycobacterial efficacy of Rubiaceae plants against fast-growing non-tuberculous *Mycobacterium* species, extracts of six South African species were tested for this particular biological activity against pathogenic *Mycobacterium* ATCC strains. Therefore, in this study, we also evaluated the anti-inflammatory activity by the inhibition of the enzyme soybean 15-LOX activity, which peroxidizes polyunsaturated fatty acids.

The MIC values of the tested extracts of each plant showed reasonable antimycobacterial activities for all strains when compared with our positive control; rifampicin. The antimycobacterial activity observed in this work could be attributed to the presence of various bioactive phytochemicals ranging from non-polar to intermediate compounds based on the phytochemical profile of the TLC plate (Supplementary Data). Our results are consistent with the study of Moraes et al. (2011); Martins et al. (2013), and Elisha et al. (2017) who reported that the antimycobacterial activity of some Rubiaceae species is related to the presence of terpenoids and alkaloids.

A diversity of bioactive natural products occurs in Rubiaceae plants. Extensive phytochemical investigations have been conducted regarding the natural occurrence of phytochemicals such as alkaloids, anthraquinones, and terpenoids in the family (Sirigiri, 2015; Sultana et al., 2015). The weak cytotoxic profile on the human macrophage cell line U937 and the human monocytic cell line THP-1 is noteworthy. These two cell lines, U937 and THP-1, are widely used to study immune response and cellular activities, either in their monocyte or macrophage-like states (Daigneault et al., 2010). However, the only difference between the U937 and THP-1 cells is in their origin, in that U937 cells are of tissue origin at more mature stage, while THP-1 cells originate from a blood leukemia origin at less mature stage, thus having the same morphology and functional properties (Liu et al., 2005; Saini et al., 2016).

Divangahi et al. (2010) showed that by activating the LOX enzyme pathway, *M. tuberculosis* not only inhibited apoptosis but also prevented cross-presentation of its antigens by dendritic cells, which impedes the initiation of T cell immunity. A drug that will interfere and prevent the activation of LOX and also inhibit apoptosis could be a good candidate for drug development. The effect of extracts from Rubiaceae plants on the activity of 15-LOX was investigated. Compared to quercetin used as positive control, extracts of *P. capensis* and *P. zombamontana* showed good inhibitory effect on LOX activity. This could imply that these extracts might have some phytochemical constituents active against LOX enzyme. LOX inhibitory activity of a Rubiaceae species (*Crossopteryx febrifuga*) was previously demonstrated by Maiga et al. (2006). The value obtained for the LOX inhibitory activity of *C. triflora* (IC_50_ = 29.86 μg/mL) also correlates with the results (IC_50_ = >50 μg/mL) of Elisha et al. (2016). Several researchers have reported on the LOX inhibitory properties of some medicinal plants and their potential for treating inflammatory diseases (Malterud and Rydland, 2000; Wangensteen et al., 2006; Ha et al., 2009).

Shah et al. (2013) reported that LOX inhibitors were involved in many inflammatory diseases and the immune response to bacterial infections. Extracts were evaluated in LPS-stimulated human macrophage cells (U937) by performing inhibition assays using six cytokines, namely, IL-2, IL-4, IL-5, IL-10, IFN-γ, and TNF-α. Monocytic cells such as U937 cells can undergo macrophage polarization depending on the type of stimulations which can either be physiological (use of IFN-γ or LPS) or exogenous chemical, phorbol esters such as phorbol-12-myristate-13-acetate (PMA). As macrophage polarization is a complex field that intersects with most physiological and pathological scenarios, M1 macrophages are typically activated by IFN-γ or LPS to produce pro-inflammatory cytokines, phagocytize microbes, and eventually initiate an immune response (Cavender et al., 1991). Activation of macrophages by LPS enhances the production of proinflammatory mediators and cytokines such as TNF-α and the IL-family (Dhar et al., 2018). Analogs of diacyl glycerol (DAG) such as PMA are known to induce protein kinase C (PKC). Hence, PMA treatment activates PKC signaling cascade in U937 to enhance the expression of a wide range of cytokines, chemokines, and genes through multiple transcription factors (Fan et al., 2014). Therefore, an M1 type macrophages are crucial for the control of immunity to intracellular mycobacteria such as TB (Murray, 2017). The assayed cytokines are commonly classified in one or the other category: TNF-α, gamma-interferon (IFN-γ), and IL-2 are pro-inflammatory cytokines whereas IL4, IL-5, and IL-10 are recognized as anti-inflammatory cytokines (Pripp and Stanišić, 2014). In Th1 responses, macrophages are activated by IFN-γ which increases pro-inflammatory and microbicidal activities (Rook, 2007). The plant extracts of *P. zombamontana* were able to induce the production of IFN-γ significantly thereby increasing microbicidal and pro-inflammatory activities in an *in vitro* model. As depicted in Figure 1, the crude extracts of *C. triflora* were able to enhance the production of TNF-α in a LPS-stimulated macrophage thereby increasing the pro-inflammatory activities. TNF-α plays a critical role in the control of mycobacteria, which drives granuloma development and also concentrates antimycobacterial drugs within macrophages (Bermudez et al., 1991).

Granuloma formation is an important mechanism necessary to inhibit the growth of mycobacteria by subjecting it to stress including starvation, reactive oxygen and nitrogen intermediates, and hypoxia, eventually restricting the replication of the bacilli (Wickremasinghe et al., 1999; O’Kane et al., 2007). However, overproduction of this important cytokine is responsible for the effects of TB including fever, weight loss, tissue necrosis, and the Koch phenomenon, i.e., the necrotic response to intradermal challenge with antigen of *M. tuberculosis* (Holland, 1996). Therefore, it is necessary to balance the release of cytokines from the Th1 and Th2 cells in order to control tumors and infections (Büssing et al., 1999). The acetone extracts of *C. natalensis*, *C. triflora*, and *P. lanceolata* enhanced the expression of IL-4 while it supressed the expression of IL-10. The amount of IL-10 produced in activated macrophages largely depends on the polarization status: M1 macrophages make IL-10, but M2 macrophages make more IL-10. Lang et al. (2002) reveal that IL-10 increases the amount of IL-4R chain on cell surface, thus making macrophages more sensitive to IL-4. All the studied extracts were able to inhibit the production of the selected Th2 cytokines and IL-5 except IL-10. It is thought that IL-10 may play an important regulatory role, preventing excessive inflammation caused by the Th1 response (Gazzinelli et al., 1992). The extracts of *C. triflora*, *O. speciosus*, and *P. zombamontana* had a higher stimulatory effect than rifampicin on IFN-γ and TNF-α production. The extract of *P. zombamontana* and *O. speciosus* enhanced the production of IL-10 despite the fact that it also had a good stimulatory effect on IFN-γ, thus revealing a mixed Th1/Th2 effect while the extracts of *C. triflora* induced a measurable degree of Th1 response. The result obtained from this study is comparable with the findings from other studies where a Th1/Th2 balance was observed for the studied plant extracts (Büssing et al., 1999; Labuschagne et al., 2013; Askari et al., 2016).

Rifampicin, a first-line anti-tubercular drug, completely inhibited the production of pro-inflammatory cytokines IFN-γ and IL-2 while stimulating the expression of anti-inflammatory IL-10. This bactericidal antibiotic therefore kills bacteria without releasing high quantities of pro-inflammatory cytokines. This result is in line with observations from other studies with rifampicin in that it suppresses the production of the pro-inflammatory cytokines and increases the expression of anti-inflammatory cytokines (Nau and Tauber, 2008). Some of these plant extracts had a higher immunomodulatory effect than the standard anti-TB drug, rifampicin, *in vitro*. According to Aro et al. (2016), the acetone extracts of *P. zombamontana* displayed good antioxidant activity (IC_50_ = 1.77 ± 0.13 μg/mL). Therefore, antioxidant substances that can scavenge and eliminate ROS may be useful in preventing or minimizing the occurrence of oxidation-related diseases such as TB (Dzoyem and Eloff, 2015). The crude extract of *P. zombamontana* therefore has promising free radical scavenging ability and stimulatory effect on macrophages to produce pro-inflammatory cytokines. The ability of the extract to induce the production of these cytokines can possibly greatly aid the immune response in combating and inhibiting the proliferation of mycobacteria.

Virulent pathogenic mycobacteria can evade the host defense by inhibiting apoptosis and triggering necrosis of host macrophages in order to delay adaptive immunity initiation (Divangahi et al., 2010). Therefore, a therapeutic agent that is able to induce apoptosis in the host macrophage can serve as a chemotherapeutic agent in combating TB. Based on the several events forming part of the apoptotic process, various techniques have been developed to detect programmed cell death. One of the early events is when cells undergoing apoptosis reorient phosphatidylserine from the inner side of the plasma membrane to its outer leaflet so cells can bind to annexin V and this process can be used as a marker for apoptosis (Schutte et al., 1998). Findings from this study suggest that the cell growth inhibition of extracts treated THP-1 cells is associated with apoptosis induction in comparison to control untreated cells in a dose and time dependent manner with the acetone extract of *C. triflora*, *P. capensis*, and *P. zombamontana* having the highest apoptotic induction. The untreated group had a low percentage of apoptotic and necrotic cells, indicating that more than 90% of the cells were viable after 24 and 48 h (Figure 4). A higher necrotic effect was observed with the treatment of *O. speciosus* and *P. lanceolata* at 50 and 100 μg/mL which are values greater than the IC_50_ values (600 and 188 μg/mL, respectively) obtained from the cytotoxicity assay. However, this discrepancies with the results between the cytotoxicity assay using MTT and annexin V/PI study could be associated to the fact that tetrazolium dye assays such as MTT are susceptible to metabolic interference and could lead to false positive results (Boyd, 1989; Haselsberger et al., 1996; Kepp et al., 2011).

Interestingly, the acetone extracts of *C. triflora* and *P. capensis* at 100 μg/mL after 48 h of treatment had the most profound apoptotic effect, resulting in a 200-fold increase. A treatment period of 48 h seems to be required to achieve an apoptosis-inducing effect as observed in this study. Study conducted by Saini et al. (2016) showed that apoptosis induction in THP-1 cells is TNF-α dependent. Therefore, the notable apoptosis induction effect exhibited by *C. triflora* could be due to its ability to induce TNF-α as observed in the immunomodulatory assessment.

While the reported apoptotic effect was observed in uninfected cells only, the data as it is, only suggests that the extracts were capable of inducing this mode of cell death which might potentially be higher in infected cells. According to a study where a gene expression analyses was done using the reverse transcription polymerase chain reaction for the iNOS and the cytokines IL-1, IL-12, IL-18, TNF-α, IFN-α, and IFN-γ in non-infected and in *Leishmania* major-infected RAW 264.7 cells, low mRNA levels was observed in non-infected cells, but considerably upregulated transcript expressions in infected cells (Radtke et al., 2004). Hence, the promising apoptotic inducing effects exhibited by these extracts could be more profound in mycobacteria-infected macrophages. This interesting preliminary result generated from this study encourages further investigations of these extracts owning to the LOX-inhibitory effect, immunomodulatory and apoptotic-inducing properties in addition to their antimycobacterial properties.

The selected extracts promoted Th1/Th2 balances in an *in vitro* model at the tested concentration which may suggest the therapeutic value of the plant in inflammatory disease such as TB. More so, any treatment or condition that favors apoptosis may have desirable effects on infections (Büssing et al., 1999). Based on the promising results generated from this study, the crude extracts of *C. triflora*, *P. capensis*, and *P. zombamontana* have the potential in an infection model to inhibit the replication of mycobacteria by stimulating the macrophages to release pro-inflammatory cytokines and scavenge free radicals such as ROS, thereby modulating the immune system to combat the disease. In addition to the above activity as possible mode of action, the extracts were able to inhibit the 15-LOX enzyme pathway in order to induce programmed cell death thereby preventing the growth of the causative organisms. By combining antimycobacterial activity, antioxidant and apoptosis induction with immunomodulatory properties, a double-edged sword can be produced to directly kill the *Mycobacterium* and boost the host immune response to indirectly help the patient in combating TB. The modulation of nitric oxide production previously reported from this plant family might contribute to their immunoregulatory and antimycobacterial effects and could be significant as an immunotherapeutic agent against TB.

## Conclusion

Findings from this study propose that some of the selected extracts have the potential to act as adjuvants in the treatment of mycobacterial infections. Further studies are required to investigate the ability of these extracts to inhibit the growth of mycobacteria intracellularly and confirm the immunological activity of these plant extracts using an infection model likewise an *in vivo* assay. Identifying bioactive principles from the acetone extract of the selected plant species could yield more leads in the battle against TB or could contribute toward standardization of herbal formulations of these plants in treating TB. These data may facilitate the development of novel chemotherapeutic agents for the management and treatment of mycobacterial infections. Isolation and identification of potential antimycobacterial compounds from the crude extracts of these plant extracts is ongoing in our laboratory.

## Author Contributions

AA, JD, and PF performed the experiments and analyzed the data. AA, JD, PF, AG, PK-K, and LM contributed to the writing of the manuscript. AA, LM, and PK-K participated in the conceptualization and supervision of the project. LM and PK-K provided funding. All authors contributed to the revision of manuscript and read and agreed on the revised version.

## Conflict of Interest Statement

The authors declare that the research was conducted in the absence of any commercial or financial relationships that could be construed as a potential conflict of interest.

## Supplementary Material

FIGURE S1 Thin layer chromatography plates developed in three mobile system **(1)** benzene/ethanol/ammonia (BEA); **(2)** chloroform/ethyl acetate/formic (CEF) and **(3)** ethyl acetate/methanol/water (EMW) sprayed with vanillin– sulphuric acid showing varied chemical constituents of the Rubiaceae plant extracts screened. PZ: *Psychotria zombamontana*, OS: *Oxyanthus speciosus*, PC: *Psychotria capensis*, PL: *Pavetta lanceolata*, CT: *Cremaspora triflora*, CN: *Cephalanthus natalensis*.

TABLE S1 Anti-mycobacterial (MIC μg/mL), cytotoxicity (IC_50_ μg/mL) and anti-lipoxygenase (IC_50_ μg/mL) activities of the tested extracts.

## Abbreviations

AA: arachidonic acid
ATCC: American Type Culture Collection
cfu: colony forming unit
COX: cyclooxygenase
DMSO: dimethyl sulfoxide
INT: iodonitrotetrazolium chloride
LOX: lipoxygenase
LPS: lipopolysaccharide
MDR: multidrug resistance
MTT: 3-(4,5-dimethyl-2-thiazolyl)-2,5-diphenyl-2H-tetrazolium bromide
PGE: prostaglandins
PMA: phorbol 12-myristate 13-acetate
TB: tuberculosis
TDR: totally drug resistance
XDR: extreme drug resistance.

## References

[B1] Anderson L. Dean A. Falzon D. (2015). *Global Tuberculosis Report 2015*, 20th Edn. Geneva: World Health Organization.

[B2] Aro A. O. Dzoyem J. P. Hlokwe T. M. Madoroba E. Eloff J. N. McGaw L. J. (2015). Some South African Rubiaceae tree leaf extracts have antimycobacterial activity against pathogenic and non-pathogenic Mycobacterium species. *Phytother. Res.* 29 1004–1010. 10.1002/ptr.5338 25857273

[B3] Aro A. O. Dzoyem J. P. McGaw L. J. (2016). In vitro synergistic antimycobacterial effect of extracts of six Rubiaceae species combined with rifampicin, and their anti-inflammatory and antioxidant activity. *BMC Complement. Altern. Med.* 16:385. 10.1186/s12906-016-1355-y 27716160 PMC5048625

[B4] Askari V. R. Rezaeeb S. A. Abnousd K. Iranshahie M. Boskabady M. H. (2016). The influence of hydro-ethanolic extract of *Portulaca oleracea L*. on Th1/Th2 balance in isolated human lymphocytes. *J. Ethnopharmacol.* 194 1112–1121. 10.1016/j.jep.2016.10.08 27842944

[B5] Behar S. M. Divangahi M. Remold H. G. (2010). Evasion of innate immunity by *Mycobacterium tuberculosis*: is death an exit strategy? *Nat. Rev. Microbiol.* 8 668–674. 10.1038/nrmicro2387 20676146 PMC3221965

[B6] Bermudez L. E. Inderlied C. Young L. S. (1991). Stimulation with cytokines enhances penetration of azithromycin into human macrophages. *Antimicrob. Agents Chemother.* 1991 2625–2629. 10.1128/AAC.35.12.2625 1667256 PMC245442

[B7] Blanco D. Perez-Herran E. Cacho M. Ballell L. Castro J. González del Rio R. (2015). *Mycobacterium tuberculosis* gyrase inhibitors (MGI) as a new class of antitubercular drugs. *J. Antimicrob. Agents Chemother.* 59 1868–1875. 10.1128/AAC.03913-14 25583730 PMC4356839

[B8] Boyd M. R. (1989). Status of the NCI preclinical antitumour drug discovery screen. *Princ. Pract. Oncol.* 3 23–42.

[B9] Büssing A. Stein G. M. Herterich-Akinpelu I. Pfuller U. (1999). Apoptosis-associated generation of reactive oxygen intermediates and release of pro-inflammatory cytokines in human lymphocytes and granulocytes by extracts from the seeds of *Acalypha wilkesiana*. *J. Ethnopharmacol.* 66 301–309. 10.1016/S0378-8741(98)00227-X 10473177

[B10] Cavender D. E. Edelbaum D. Welkovich L. (1991). Effects of inflammatory cytokines and phorbol esters on the adhesion of U937 cells, a human monocyte-like cell line, to endothelial cell monolayers and PLoS extracellular matrix proteins. *J. Leukoc. Biol.* 49 566–578. 10.1002/jlb.49.6.566 1673992

[B11] Cho J. H. (2008). The genetics and immuno pathogenesis of inflammatory bowel disease. *Nat. Rev. Immunol.* 8 458–466. 10.1038/nri2340 18500230

[B12] Choudhury K. T. Choudhury M. D. Baruah M. (2012). Anti-bacterial activity of some plants belonging to the family Rubiaceae: a review. *World J. Pharm. Sci.* 1 1179–1194.

[B13] Daigneault M. Preston J. A. Marriott H. M. Whyte M. K. Dockrell D. H. (2010). The identification of markers of macrophage differentiation in PMA-stimulated THP-1 cells and monocyte-derived macrophages. *PLoS One* 5:e8668. 10.1371/journal.pone.0008668 20084270 PMC2800192

[B14] Devasagayam T. P. Sainis K. B. (2002). Immune system and antioxidants, especially those derived from Indian medicinal plants. *Indian J. Exp. Biol.* 40 639–655. 12587713

[B15] Dhar R. Kimseng R. Chokchaisiri R. Hiransai P. Utaipan T. Suksamrarn A. (2018). 2’,4-Dihydroxy-3’,4’,6’-trimethoxychalcone from Chromolaena odorata possesses anti- inflammatory effects via inhibition of NF-κB and p38 MAPK in lipopolysaccharide-activated RAW 264.7 macrophages. *Immunopharmacol. Immunotoxicol.* 40 43–51. 10.1080/08923973.2017.1405437 29199487

[B16] Divangahi M. Behar S. M. Remold H. (2013). Dying to live: how the death modality of the infected macrophage affects immunity to tuberculosis. *Adv. Exp. Med. Biol.* 783 103–120. 10.1007/978-1-4614-6111-1_6 23468106 PMC4678885

[B17] Divangahi M. Desjardins D. Nunes-Alves C. Remold H. G. Behar S. M. (2010). Eicosanoid pathways regulate adaptive immunity to Mycobacterium tuberculosis. *Nat. Immunol.* 11 751–758. 10.1038/ni.1904 20622882 PMC3150169

[B18] Du C. Y. Q. Choi R. C. Y. Zheng K. Y. Z. Dong T. T. X. Lau D. T. W. Tsim K. W. K. (2013). Yu Ping Feng San, an ancient Chinese herbal decoction containing Astragali Radix, Atractylodis Macrocephalae Rhizoma and Saposhnikoviae Radix, regulates the release of cytokines in murine macrophages. *PLoS One* 8:e78622. 10.1371/journal.pone.0078622 24244327 PMC3823765

[B19] Dzoyem J. P. Eloff J. N. (2015). Anti-inflammatory, anticholinesterase and antioxidant activity of leaf extracts of twelve plants used traditionally to alleviate pain and inflammation in South Africa. *J. Ethnopharmacol.* 160 194–201. 10.1016/j.jep.2014.11.034 25476488

[B20] Elisha I. L. Botha F. S. Madikizela B. McGaw L. J. Eloff J. N. (2017). Acetone leaf extracts of some South African trees with high activity against *Escherichia coli* also have good antimycobacterial activity and selectivity index. *BMC Complement. Alter. Med.* 17:327. 10.1186/s12906-017-1831-z 28629354 PMC5477271

[B21] Elisha I. L. Dzoyem J. P. McGaw L. J. Botha F. S. Eloff J. N. (2016). The anti-arthritic, anti-inflammatory, antioxidant activity and relationships with total phenolics and total flavonoids of nine South African plants used traditionally to treat arthritis. *BMC Complement. Alter. Med.* 16:307. 10.1186/s12906-016-1301-z 27554099 PMC4995646

[B22] Eloff J. N. (1998). A sensitive and quick microplate method to determine the minimum inhibitory concentration of plant extracts for bacteria. *Planta Med.* 64 711–714. 10.1055/s-2006-957563 9933989

[B23] Etna M. P. Giacomini E. Severa M. Coccia E. M. (2014). Pro- and anti-inflammatory cytokines in tuberculosis: a two-edged sword in TB pathogenesis. *Semin. Immunol.* 26 543–551. 10.1016/j.smim.2014.09.011 25453229

[B24] Fan H. C. Fernandez-Hernando C. Lai J. H. (2014). Protein kinase C isoforms in atherosclerosis: pro- or anti- inflammatory? *Biochem. Pharmacol.* 88 139–149. 10.1016/j.bcp.2014.01.006 24440741

[B25] Fratazzi C. Arbeit R. D. Carini C. Remold H. G. (1997). Programmed cell death of *Mycobacterium avium* serovar 4-infected human macrophages prevents the mycobacteria from spreading and induces mycobacterial growth inhibition by freshly added, uninfected macrophages. *J. Immunol.* 158 4320–4327. 9126994

[B26] Gazzinelli R. T. Oswald I. P. James S. L. Sher A. (1992). IL-10 inhibits parasite killing and nitrogen oxide production by IFN-gamma-activated macrophages. *J. Immunol.* 148 1792–1796. 1541819

[B27] Ha D. T. Kim H. Thuong P. T. Ngoc T. M. Lee I. Hung N. D. (2009). Antioxidant and lipoxygenase inhibitory activity of oligostilbenes from the leaf and stem of *Vitis amurensis*. *J. Ethnopharmacol.* 125 304–309. 10.1016/j.jep.2009.06.019 19560532

[B28] Haselsberger K. Peterson D. C. Thomas D. G. Darling J. L. (1996). Assay of anticancer drugs in tissue culture: comparison of a tetrazolium-based assay and a protein binding dye assay in short term cultures derived from human malignant glioma. *Anticancer Drugs* 7 331–338. 10.1097/00001813-199605000-00014 8792008

[B29] Holland S. M. (1996). Therapy of mycobacterial infections. *Res. Immunol.* 147 572–581. 10.1016/S0923-2494(97)85224-8 9127890

[B30] Hou C. C. Chen C. H. Yang N. S. Chen Y. P. Lo C. P. Wang S. Y. (2010). Comparative metabolomics approach coupled with cell- and gene-based assays for species classification and anti- inflammatory bioactivity validation of Echinacea plants. *J. Nutr. Biochem.* 21 1045–1059. 10.1016/j.jnutbio.2009.08.010 20005088

[B31] Kepp O. Galluzzi L. Lipinski M. Yuan J. Kroemer G. (2011). Cell death assays for drug discovery. *Nat. Rev. Drug Discov.* 10 221–237. 10.1038/nrd3373 21358741

[B32] Kleinnijenhuis J. Oosting M. Joosten L. A. Netea M. G. van Crevel R. (2011). Innate immune recognition of *Mycobacterium tuberculosis*. *Clin. Dev. Immunol.* 2011:405310. 10.1155/2011/405310 21603213 PMC3095423

[B33] Kotzé M. Eloff J. N. Houghton P. J. (2002). Extraction of antibacterial compounds from *Combretum microphyllum* (Combretaceae). *S. Afr. J. Bot.* 68 62–67. 10.1016/S0254-6299(15)30442-7

[B34] Kuete V. (2010). Potential of Cameroonian plants and derived natural products against microbial infections; a review. *Planta Med.* 76 1479–1491. 10.1055/s-0030-1250027 20533165

[B35] Labuschagne C. F. van den Broek N. J. Postma P. Berger R. Brenkman A. B. (2013). A protocol for quantifying lipid peroxidation in cellular systems by F2-isoprostane analysis. *PLoS One* 8:e80935. 10.1371/journal.pone.0080935 24244726 PMC3828286

[B36] Lang R. Patel D. Morris J. J. Rutschman R. L. Murray P. J. (2002). Shaping gene expression in activated and resting primary macrophages by IL-10. *J. Immunol.* 169 2253–2263. 10.4049/jimmunol.169.5.2253 12193690

[B37] Liu H. Z. Gong J. P. Wu C. X. Peng Y. Li X. H. You H. B. (2005). The U937 cell line induced to express CD14 protein by 1,25-dihydroxyvitamin D3 and be sensitive to endotoxin stimulation. *Hepatobiliary Pancreat. Dis. Int.* 4 84–89. 15730927

[B38] Maiga A. Malterud K. E. Diallo D. Paulsen B. S. (2006). Antioxidant and 15-lipoxygenase inhibitory activities of the Malian medicinal plants *Diospyros abyssinica* (Hiern) F. White (Ebenaceae), *Lannea velutina* A. Rich (Anacardiaceae) and *Crossopteryx febrifuga* (Afzel) Benth. (Rubiaceae). *J Ethnopharmacol.* 104 132–137. 10.1016/j.jep.2005.08.063 16213686

[B39] Malterud K. E. Rydland K. M. (2000). Inhibitors of 15-lipoxygenase from orange peel. *J. Agric. Food Chem.* 48 5576–5580. 10.1021/jf000613v 11087521

[B40] Martins D. Carrion L. L. Ramos D. F. Salomé K. S. da Silva P. E. Barison A. (2013). Triterpenes and the antimycobacterial activity of Duroia macrophylla Huber (Rubiaceae). *Biomed. Res. Int.* 2013:605831. 10.1155/2013/605831 23509750 PMC3583080

[B41] McGaw L. J. Lall N. Meyer J. J. M. Eloff J. N. (2008). The potential of South African plants against Mycobacterium infections. *J. Ethnopharmacol.* 119 482–500. 10.1016/j.jep.2008.08.022 18805475

[B42] McGaw L. J. Steenkamp V. Eloff J. N. (2007). Evaluation of *Athrixia* bush tea for cytotoxity, antioxidant activity, caffeine content and presence of pyrrolizidine alkaloids. *J. Ethnopharmacol.* 110 16–22. 10.1016/j.jep.2006.08.029 17045437

[B43] Molloy A. Laochumroonvorapong P. Kaplan G. (1994). Apoptosis, but not necrosis, of infected monocytes is coupled with killing of intracellular bacillus Calmette-Guerin. *J. Exp. Med.* 180 1499–1509. 10.1084/jem.180.4.1499 7931080 PMC2191680

[B44] Moraes T. M. de Araújo M. H. Bernardes N. R. de Oliveira D. B. Lasunskaia E. B. Muzitano M. F. (2011). Antimycobacterial activity and alkaloid prospection of Psychotria species (Rubiaceae) from the Brazilian Atlantic Rainforest. *Planta Med.* 77 964–970. 10.1055/s-0030-1250656 21243585

[B45] Mosmann T. (1983). Rapid colorimetric assay for cellular growth and survival: application to proliferation and cytotoxicity. *J. Immunol. Methods* 65 55–63. 10.1016/0022-1759(83)90303-4 6606682

[B46] Murray P. J. (2017). Macrophage Polarization. *Annu. Rev. Physiol.* 79 542–566. 10.1146/annurev-physiol-022516-034339 27813830

[B47] Nau R. Tauber S. C. (2008). Immunomodulatory properties of Antibiotics. *Curr. Mol. Pharmacol.* 1 68–79. 10.2174/1874467210801010068 20021425

[B48] Nguta J. M. Appiah-Opong R. Nyarko A. K. Yeboah-Manu D. Addo G. A. (2015). Current perspectives in drug discovery against tuberculosis from natural products. *Int. J. Mycobacteriol.* 4 165–183. 10.1016/j.ijmyco.2015.05.004 27649863

[B49] Ngwenya N. M. (2008). *Biological and Phytochemical Screening of Major Compounds in Cephalanthus natalensis. Ph. D. thesis*, University of Johannesburg, Johannesburg.

[B50] Nowakowska Z. (2007). A review of anti-infective and anti-inflammatory chalcones. *Eur. J. Med. Chem.* 42 125–137. 10.1016/j.ejmech.2006.09.019 17112640

[B51] O’Kane C. M. Boyle J. J. Horncastle D. E. Elkington P. T. Friedland J. S. (2007). Monocyte- dependent fibroblast CXCL8 secretion occurs in tuberculosis and limits survival of mycobacteria within macrophages. *J. Immunol.* 178 3767–3776. 10.4049/jimmunol.178.6.3767 17339475

[B52] Ouchi N. Parker J. L. Lugus J. J. Walsh K. (2011). Adipokines in inflammation and metabolic disease. *Nat. Rev. Immunol.* 11 85–97. 10.1038/nri2921 21252989 PMC3518031

[B53] Passmore J. S. Lukey P. T. Ress S. R. (2001). The human macrophage cell line U937 as an *in vitro* model for selective evaluation of mycobacterial antigen-specific cytotoxic T-cell function. *Immunology* 102 146–156. 10.1046/j.1365-2567.2001.01164.x 11260319 PMC1783164

[B54] Pinto M. D. C. Tejeda A. Duque A. L. Macias P. (2007). Determination of lipoxygenase activity in plant extracts using a modified ferrous oxidation-xylenol orange assay. *J. Agric. Food Chem.* 55 5956–5959. 10.1021/jf070537x 17602650

[B55] Pripp A. H. Stanišić M. (2014). The correlation between pro- and anti-inflammatory cytokines in chronic subdural hematoma patients assessed with factor analysis. *PLoS One* 9:e90149. 10.1371/journal.pone.0090149 24587250 PMC3937441

[B56] Radtke O. A. Kiderlen A. F. Kayser O. Kolodziej H. (2004). Gene expression profiles of inducible nitric oxide synthase and cytokines in Leishmania major-Infected Macrophage-Like RAW 264.7 cells treated with gallic Acid. *Planta Med.* 70 924–928. 10.1055/s-2004-832618 15490320

[B57] Rayasam G. V. Balganesh T. S. (2015). Review: exploring the potential of adjunct therapy in tuberculosis. *Trends Pharmacol. Sci.* 36 506–513. 10.1016/j.tips.2015.05.005 26073420

[B58] Riccardi G. Pasca M. R. (2014). Trends in discovery of new drugs for tuberculosis therapy. *J. Antibiot.* 67 655–659. 10.1038/ja.2014.109 25095807

[B59] Rook G. A. W. (2007). Th2 cytokines in susceptibility to tuberculosis. *Curr. Mol. Med.* 7 327–337. 10.2174/156652407780598557 17504117

[B60] Saini N. K. Sinha R. Singh P. Sharma M. Pathak R. Rathor N. (2016). Mce4A protein of *Mycobacterium tuberculosis* induces pro-inflammatory cytokine response leading to macrophage apoptosis in a TNF-a dependent manner. *Microb. Pathog.* 100 43–50. 10.1016/j.micpath.2016.08.038 27592091

[B61] Schutte B. Nuydens R. Geerts H. Ramaekers F. (1998). Annexin V binding assay as a tool to measure apoptosis in differentiated neuronal cells. *J. Neurosci. Methods* 86 63–69. 10.1016/S0165-0270(98)00147-2 9894786

[B62] Shah S. M. A. Ashraf M. Ahmad I. Arshad S. Yar M. Latif A. (2013). Anti-lipoxygenase activity of some indigenous medicinal plants. *J. Med. Plant Res.* 7 219–222. 10.5897/JMPR11.194 19701137

[B63] Sirigiri C. K. (2015). Medicinal attributes of family Rubiaceae. *Int. J. Pharm. Biol. Sci.* 5 179–181. 27899258

[B64] Sultana N. Islam T. de Alencar M. V. O. B. Silva S. W. C. Chowdhury M. U. Melo-Cavalcante A. A. C. (2015). Phyto-pharmacological screenings of two Rubiaceae family plants. *Afr. J. Pharm. Pharmacol.* 9 775–782. 10.5897/AJPP2015.4313

[B65] Wangensteen H. Miron A. Alamgir M. Rajia S. Samuelsen A. B. Malterud K. E. (2006). Antioxidant and 15-lipoxygenase inhibitory activity of rotenoids, isoflavones and phenolic glycosides from *Sarcolobus globosus*. *Fitoterapia* 77 290–295. 10.1016/j.fitote.2006.03.017 16701962

[B66] Wickremasinghe M. I. Thomas L. H. Friedland J. S. (1999). Pulmonary epithelial cells are a source of IL-8 in the response to *Mycobacterium tuberculosis*: essential role of IL-1 from infected monocytes in a NF-kappa B-dependent network. *J. Immunol.* 163 3936–3947. 10490995

[B67] World Health Organization [WHO] (2017). *Global Tuberculosis Report 2017.* Geneva: World Health Organization.

[B68] Worthington R. J. Melander C. (2013). Combination approaches to combat multidrug-resistant bacteria. *Trends Biotechnol.* 31 177–184. 10.1016/j.tibtech.2012.12.006 23333434 PMC3594660

