# Supplementary figures and images for: In vitro Antimycobacterial, Apoptosis-Inducing Potential, and Immunomodulatory Activity of Some Rubiaceae Species
Source: Front Pharmacol. 2019 Mar 5;10:185. doi: 10.3389/fphar.2019.00185 (PMC6413436; doi:10.3389/fphar.2019.00185)

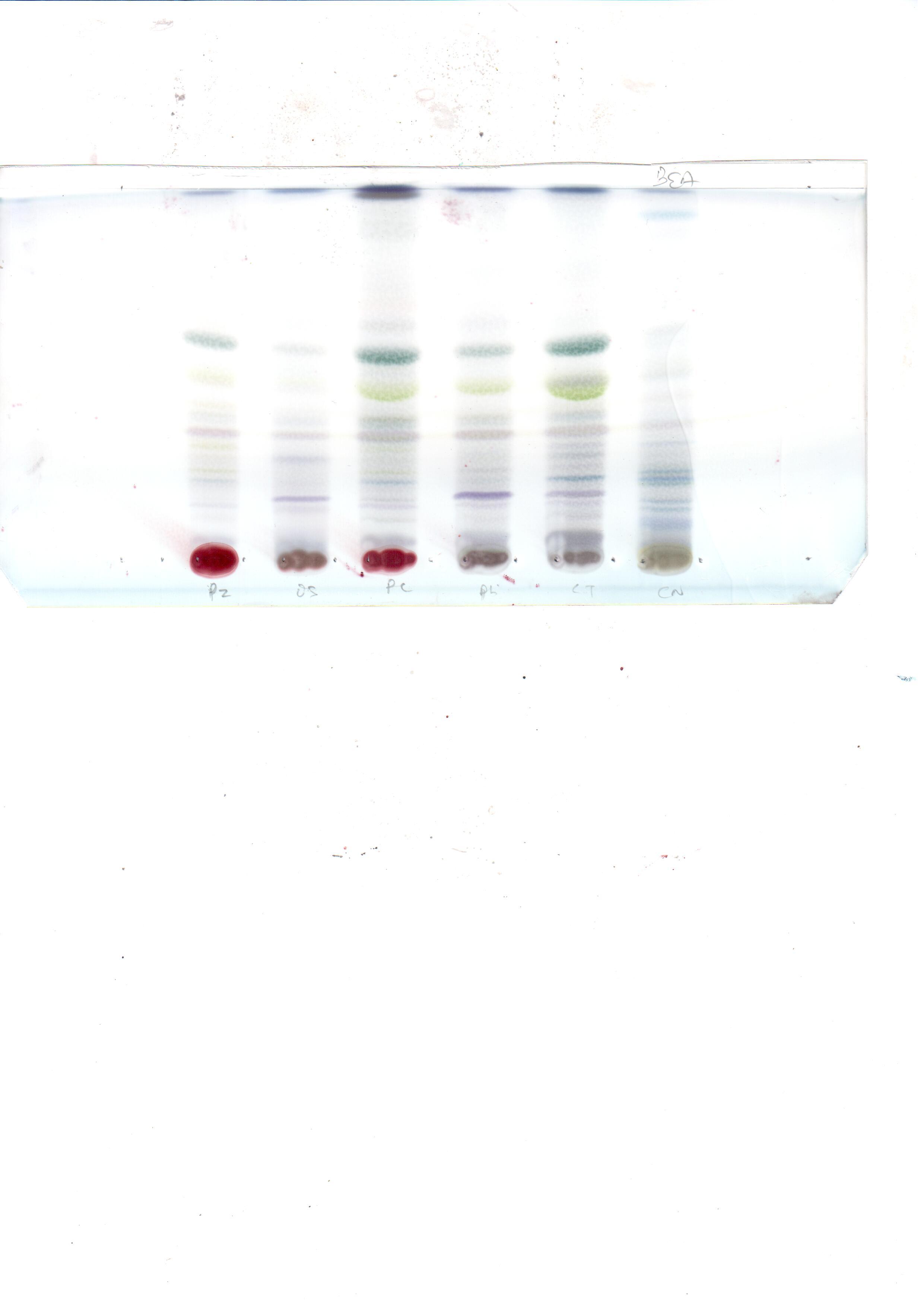

Supplement: FIGURE S1 — Thin layer chromatography plates developed in three mobile system (1) benzene/ethanol/ammonia (BEA); (2) chloroform/ethyl acetate/formic (CEF) and (3) ethyl acetate/methanol/water (EMW) sprayed with vanillin– sulphuric acid showing varied chemical constituents of the Rubiaceae plant extracts screened. PZ: Psychotria zombamontana, OS: Oxyanthus speciosus, PC: Psychotria capensis, PL: Pavetta lanceolata, CT: Cremaspora triflora, CN: Cephalanthus natalensis. [file Image_1.JPEG]

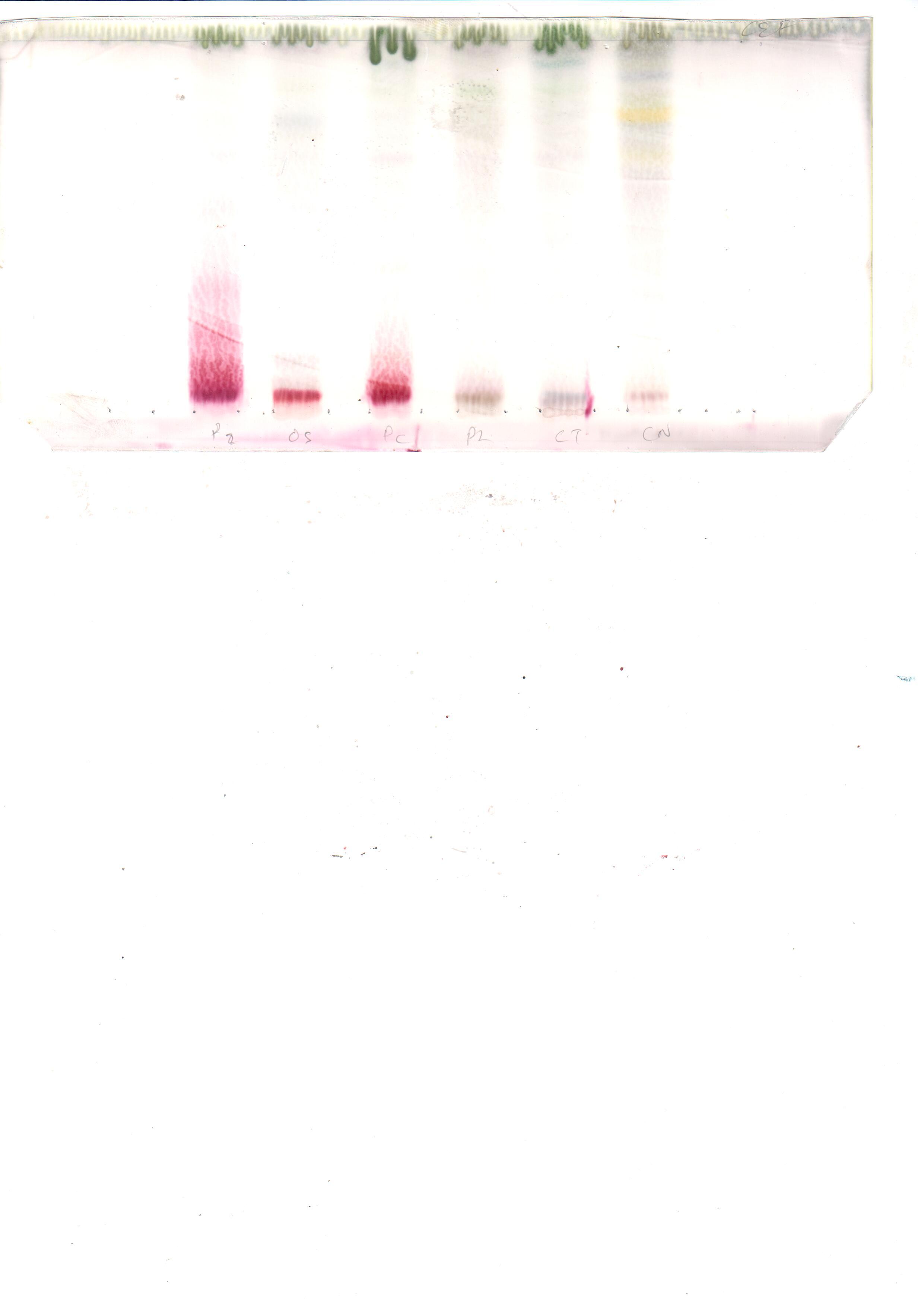

Supplement: Supplementary file 2 [file Image_2.JPEG]

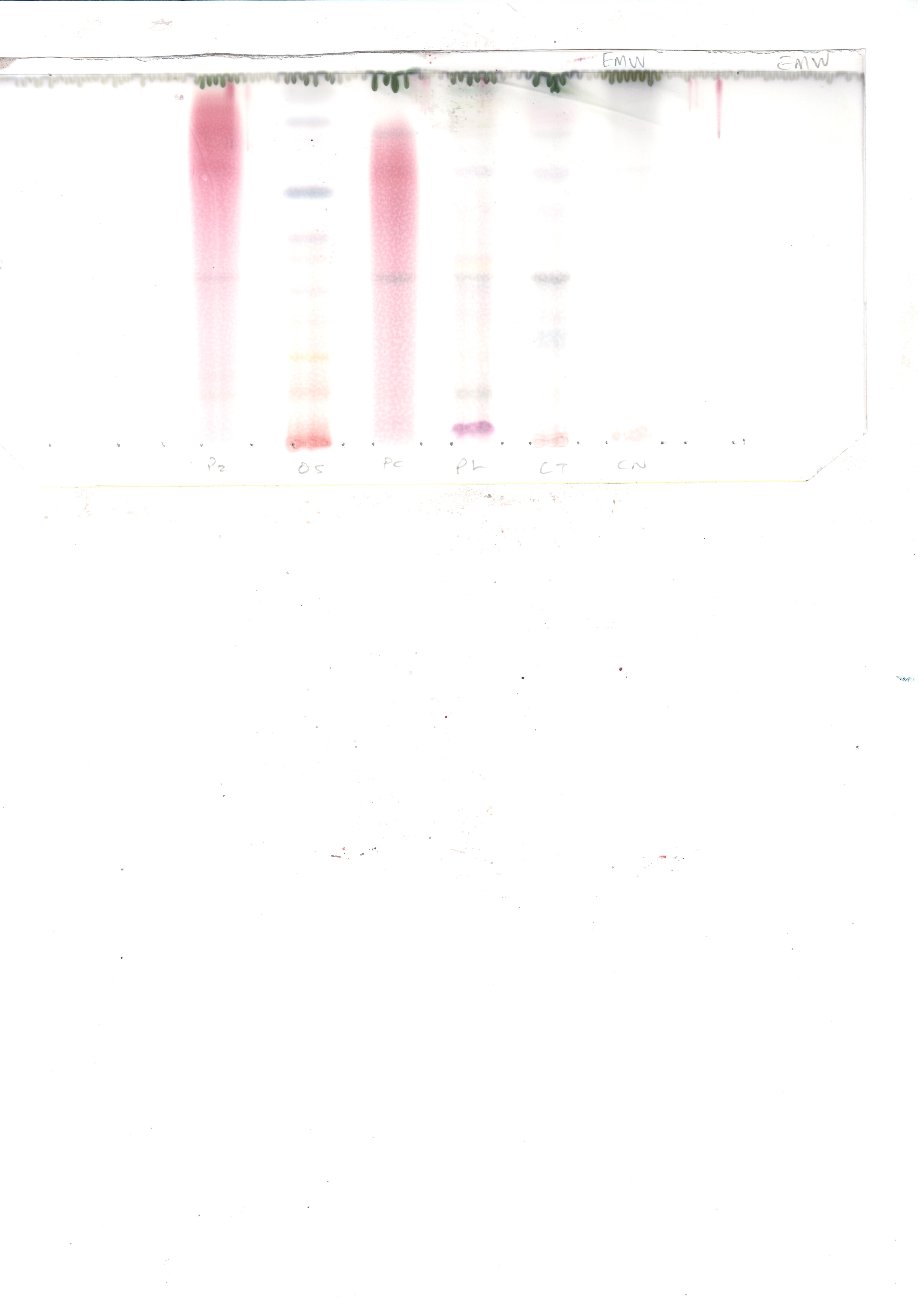

Supplement: Supplementary file 3 [file Image_3.JPEG]
