# Supplementary material for: In vitro Antimycobacterial, Apoptosis-Inducing Potential, and Immunomodulatory Activity of Some Rubiaceae Species
Source: Front Pharmacol. 2019 Mar 5;10:185. doi: 10.3389/fphar.2019.00185 (PMC6413436; doi:10.3389/fphar.2019.00185)
Supplement: TABLE S1 — Anti-mycobacterial (MIC μg/mL), cytotoxicity (IC50 μg/mL) and anti-lipoxygenase (IC50 μg/mL) activities of the tested extracts. [file Table_1.DOCX]

**TABLE 1** Anti-mycobacterial (MIC µg /mL**)**, cytotoxicity (IC_50_ µg /mL) and anti-lipoxygenase (IC_50_ µg /mL) activities of the tested extracts.

| **Samples** | **MIC (µg /mL)** | | | **IC_50_** | | | |
| --- | --- | --- | --- | --- | --- | --- | --- |
|  | ***H37Rv*** | ***M. tb*** | ***M. bovis*** | **U937** | **THP-1** | | **LOX** |
| *Cephalanthus natalensis* Oliv. | **78** | 156 | 156 | 100 ± 0.01 | | 100 ± 0.01 | 32.45 ± 1.05 |
| *Cremaspora triflora* Thonn. | 156 | **39** | 156 | 100 ± 0.10 | | 378 ± 0.21 | 29.86 ± 2.39 |
| *Oxyanthus speciosus* DC. | **78** | **78** | 312 | 190 ± 0.00 | | 600 ± 0.01 | 11.20 ± 7.30 |
| *Pavetta lanceolata* Eckl. | 156 | 156 | **78** | 125 ± 0.36 | | 188 ± 0.03 | 10.85 ± 0.83 |
| *Psychotria capensis* Vatke | 156 | 312 | 156 | **25** **± 0.00** | | **761 ± 0.10** | 5.8 ± 1.94 |
| *Psychotria zombamontana* Kuntze | 156 | 312 | **78** | 400 ± 0.00 | | 579 ± 0.57 | 4.32 ± 1.10 |
| Rifampicin | 0.05 | 0.07 | 0.19 | >200 ± 0.02 | | nd | nd |
| Streptomycin | 0.1 | 0.5 | 0.1 | nd | | nd | nd |
| Puromycin | nd | nd | nd | 4 ± 0.02 | | 3.6 ± 0.01 | nd |
| Quercetin | nd | nd | nd | nd | | nd | 25.53 ± 1.18 |

nd: not determined; LOX: 15-lipoxygenase
